# Difficulties in Interpreting IGF-1 Levels in Short Stature Children Born Small for Gestational Age (SGA) Treated with Recombinant Human Growth Hormone (rhGH) Based on Data from Six Clinical Centers in Poland

**DOI:** 10.3390/jcm12134392

**Published:** 2023-06-29

**Authors:** Marta Glińska, Mieczysław Walczak, Beata Wikiera, Beata Pyrżak, Anna Majcher, Monika Paluchowska, Aneta Gawlik, Aleksandra Antosz, Marcin Kusz, Artur Bossowski, Karolina Stożek, Anna Wędrychowicz, Jerzy Starzyk, Elżbieta Petriczko

**Affiliations:** 1 Department of Paediatrics, Endocrinology, Diabetology, Metabolic Disorders and Cardiology of the Developmental Age, Pomeranian Medical University, 71-252 Szczecin, Poland; mieczyslaw.walczak@pum.edu.pl; 2 Department of Endocrinology and Diabetology of Children and Adolescents, Wroclaw Medical University, 50-367 Wroclaw, Poland; kep@usk.wroc.pl; 3 Department of Paediatrics and Endocrinology, Medical University of Warsaw, 02-091 Warsaw, Poland; endokrynologia.dsk@uckwum.pl (B.P.); amajcher@wum.edu.pl (A.M.); mpaluchowska@wum.edu.pl (M.P.); 4 Department of Paediatrics and Paediatric Endocrinology with Division of Sex Development Disorders, Upper Silesia Children’s Health Centre, Medical University of Silesia, 40-055 Katowice, Poland; endo_sk6@sum.edu.pl (A.G.); aantosz@sum.edu.pl (A.A.); marcinkusz.mk@gmail.com (M.K.); 5 Department of Paediatrics, Endocrinology, Diabetology with Cardiology Division, Medical University of Bialystok, 15-089 Bialystok, Poland; abossowski@hotmail.com (A.B.); 2klchdz@umb.edu.pl (K.S.); 6 Department of Endocrinology of Children and Young Adults, Collegium Medicum, Jagiellonian University, 31-008 Krakow, Poland; endodim@cm-uj.krakow.pl (A.W.); jerzy.starzyk@uj.edu.pl (J.S.)

**Keywords:** SGA, IGF-1, recombinant human growth hormone

## Abstract

The assessment of IGF-1 concentrations is one of the parameters used for evaluating response to rhGH treatment. An increase in IGF-1 concentration positively correlates with growth improvement, whereas IGF-1 concentrations significantly above the reference range may increase the risk of possible side effects. The aim of this study was to evaluate the IGF-1 local reference ranges for the rhGH treatment centers concerned and to compare these values with the population reference ranges. A retrospective analysis was conducted on auxological data from 229 SGA patients who received rhGH treatment between 2016 and 2020 at six university clinical centers in Poland. The IGF-1 levels were assessed at baseline, after 12 and 24 months, and compared to the reference ranges provided by the local laboratory and to the population reference ranges. After 12 months, 56 patients (24%) presented IGF-1 values > 97th percentile for the local reference range, whereas only 8 (3.5%) did so using the population reference ranges; *p* < 0.001. After 24 months of treatment, the values were: 47 (33%) > 97th percentile by local vs. 6 (4.2%) by population standards; *p* < 0.001. Thirty-nine patients had rhGH dose reduced after 12 months, of whom twelve (25%) had IGF-1 > 97th percentile according to the local reference ranges and five (13%) > 97th percentile for the population. Our data suggest that different methods used to determine IGF-1 concentration and the different IGF-1 reference ranges result in a significant proportion of rhGH-treated children with elevated IGF-1 concentration and experiencing dose reductions, which may negatively affect growth rate.

## 1. Introduction

For many years, patients born small for gestational age (SGA) have been qualified for growth hormone therapy. SGA is defined as a birth weight and/or length that is at least two standard deviations (SDs) below the mean for gestational age in the population [1].

Within the first 6–12 months of life, the majority of children born SGA experience accelerated growth and reach the population norm. However, around 10% of these children do not catch up and need additional diagnostic and therapeutic interventions [2]. Increasing evidence suggests that an adverse intrauterine environment and rapid postnatal weight gain in young SGA children contribute to an increased risk of developing chronic diseases in adulthood, such as type 2 diabetes mellitus or metabolic syndrome, as well as a short stature [3].

Growth hormone treatment programs have been developed for these children, both to improve height and the metabolic condition of the patients. However, it should be kept in mind that SGA patients form a heterogeneous group, whose common factor is a birth weight/length deficiency. Therefore, these patients in particular require an individualized therapy.

The assessment of insulin-like growth factor 1 (IGF-1) concentrations is one of the most important parameters for evaluating response to rhGH treatment [4]. It has been shown that an increase in IGF-1 concentration positively correlates with growth improvement [5,6]. On the other hand, IGF-1 concentrations significantly above the reference range may increase the risk of atherosclerosis or malignancy [7]. By optimizing the dosage, patients can achieve maximum gains in height, body composition, and metabolic outcomes, while minimizing the occurrence of adverse events associated with rhGH treatment [6,8,9].

The assessment of IGF-1 levels is listed as one of the mandatory tests performed during routine follow-up visits in patients treated with rhGH [10]. Modification of rhGH dosage depends on IGF-1 values. At the same time, documented persistently elevated IGF-1 levels above the age- and sex-specific norm are an indication for temporary withholding or termination of treatment [11]. During the previous analysis of the results of the response to treatment of children born small for gestational age (SGA) in six clinical centers in Poland, it was noted that a high percentage of children required modification of the growth hormone dose or early termination of treatment [12]. At the same time, IGF-1 concentration levels maintained within the normal range in accordance to IGF-1 ranges were provided by Bedogni et al. [13]. This finding has raised interest in understanding why so many patients in daily clinical practice require modification of their growth hormone dose.

Insulin-like growth factor 1 is a small protein that consists of two chains, A and B, linked by disulfide bridges, and a C-peptide of 12 amino acids. Its synthesis is encoded by the *IGF1* gene located on the long arm of chromosome 12, locus 12q22-23 [14]. The vast majority of IGF-1 is produced in the liver by a growth hormone-dependent mechanism. The primary mechanism of action of IGF-1 is endocrine. A significant proportion of tissues in the body also produce IGF-1, which acts auto- and paracrine at the site of secretion, such as in the growth plates of long bones [15].

IGF-1 exerts mitogenic and anabolic effects, promotes cell proliferation and differentiation, is a stimulator of neuroblast migration and protein and sex steroid synthesis, and exhibits anti-apoptotic effects. Its concentration changes at different stages of life—it increases from infancy to puberty, after which it gradually decreases [16]. When interpreting results, it is crucial to consider various physiological variables and clinical conditions that can influence the concentrations of IGF-1 [17]. Factors such as age, pubertal stage, pregnancy, and extremes of body mass index hold particular significance and must be taken into account [18].

The vast majority of IGF-1 (up to 99%) is present in the body’s circulation in the form bound to insulin-like growth factor binding proteins (IGFBP1–6) and about 80% in the form of a ternary complex, bound to IGFBP-3 and the acid-labile subunit (ALS) [19]. The complex stabilizes growth hormone, retains it longer in the blood plasma, and thus aids GH in reaching the target tissues [20].

The GH complex, in association with IGFBP-3 and ALS, exhibits a half-life ranging from 12 to 15 h. In contrast, GH is secreted in a pulsatile manner and has a shorter blood half-life of 20–50 min [21,22]. Recognizing the greater stability of IGF-1 secretion throughout the day, it is now widely acknowledged that IGF-1 serves as a superior marker for evaluating treatment response and patient compliance compared to GH [23].

All of the above need to be taken into account while choosing the proper laboratory assay and providing best timing for IGF-1 assessment in order to avoid prelaboratory and laboratory error.

The aim of this work was to evaluate the reference values and IGF-1 norms for the rhGH treatment centers concerned, to compare these values with the population norm (using the reference values presented by Bedogni et al.) [12] and to assess the need for modification of the growth hormone dose based on the different norms.

## 2. Materials and Methods

### 2.1. Study Design

The nature of the study was observational, and it involved collecting and retrospectively analyzing auxological data from patients who received rhGH treatment between 2016 and 2020 at six university clinical centers (Szczecin, Katowice, Wroclaw, Krakow, Warsaw, and Bialystok).

Short stature children with SGA who meet the unified guidelines are eligible for participation in the Polish rhGH treatment program. The inclusion criteria for this program include: short stature defined by Ht < −2 SDs and ΔHV < −1 SD based on Polish population norms; age above 4 years; GH concentration ≥ 10 ng/mL, as determined by two out of four growth hormone secretion stimulation tests (with clonidine, L-Dopa, arginine, or insulin), or by a nocturnal growth hormone secretion test (with at least five GH measurements); birth weight or length <−2 SD for gestational age and sex according to population norms; BA < 14 years in girls and BA < 16 years in boys (assessed using the Greulich–Pyle method); exclusion of contraindications to GH therapy through contrast-enhanced CT or MRI of the hypothalamic–pituitary region, as well as exclusion of other causes of short stature. All of these criteria must be met. Karyotype testing is required for all short stature girls before treatment initiation.

Once enrolled in the rhGH program, patients are required to attend follow-up visits initially every 3 months during the first year of treatment and later every 6 months. These visits include standardized tests such as anthropometric measurements, laboratory tests, and BA assessment. For the purpose of this study, data from the qualification process, as well as data from routine follow-up visits at 12 and 24 months (±3 months), were collected using a nationwide monitoring system.

The IGF-1 levels were assessed in every patient at baseline and after 12 and 24 months of treatment. The fasting samples were tested in every treatment center within 24 h from blood withdrawal.

The IGF-1 assay values collected at a specific center were compared to the norms provided by the local laboratory, adapted to the method used for the assay. The patients from Szczecin and Bialystok were combined into a single subgroup due to the use of the exact same method for determining IGF-1 levels (Elecsys^®^ IGF-1, Roche Diagnostics International Ltd., Rotkreuz, Switzerland) within the same laboratory network as well as the same IGF-1 values.

The criteria for discontinuing GH treatment are as follows: femoral head exfoliation, pseudo-tumor cerebri, diabetes mellitus, diagnosis or recurrence of proliferative disease, lack of consent from the patient (or legal guardian) to continue treatment or poor compliance, unsatisfactory treatment effect defined as ΔHV < 2 cm/year, BA > 14 years for girls and BA > 16 years for boys, significantly worsened body proportion disorders, major congenital malformations affecting essential vital functions, chromosomal abnormalities associated with an increased risk of proliferative diseases, and elevated IGF-1 levels relative to age and sex observed 3 months after discontinuation of growth hormone therapy.

All patients within our study group met the qualification requirements for rhGH treatment in accordance with the Polish guidelines. The inclusion criteria for our study consisted of the following: short stature defined as Ht < 3rd percentile or Ht < −2 SDs based on the Polish population norms, diagnosis of SGA indicated by birth weight or length <−2 SD for gestational age and sex according to population norms, and a minimum duration of 12 months of growth hormone treatment. The exclusion criteria encompassed advanced puberty during treatment and poor compliance.

As there is no population-based standard for IGF-1 concentrations in Poland at the time of writing the previous study involving the same group of patients, in order to standardize the results, the norms presented by Bedogni et al. were used [12]. The Italian study population consisted of 24,403 healthy children aged 0 to 18 years, which made it possible to develop reliable norms for the pediatric population.

The aim of this study was to evaluate the IGF-1 concentration in patients treated with rhGH and comparison of the results with norms provided by the local laboratory and a general norm based on the Italian norm presented by Bedogni et al. [13].

**Figure 1:**
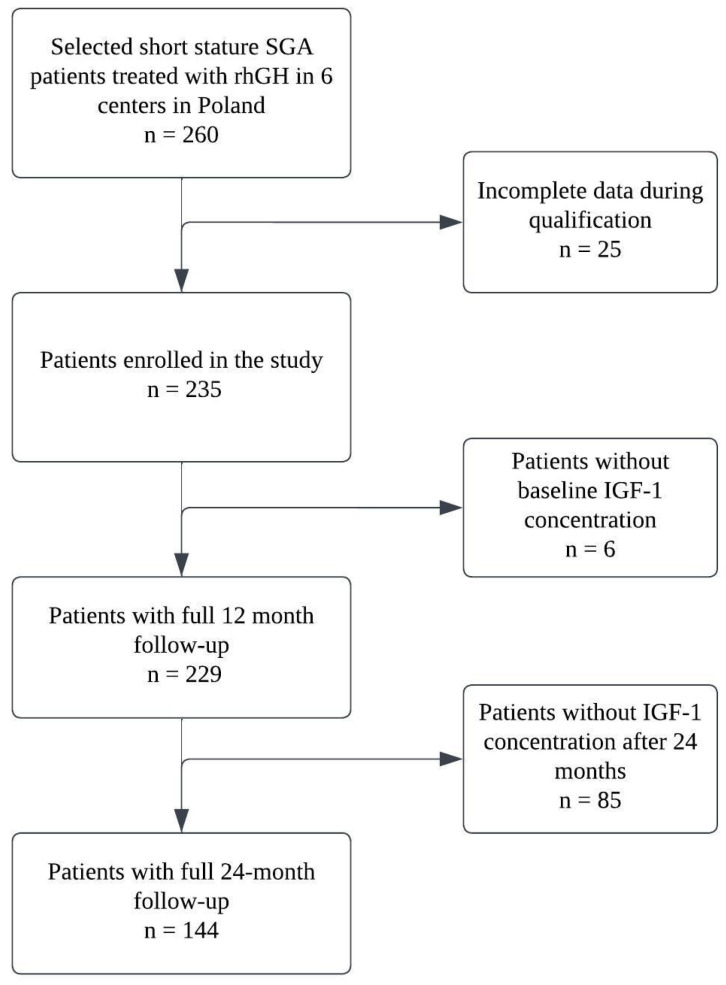
Data collection process. SGA—small for gestational age; rhGH—recombinant human growth hormone; IGF-1—insulin-like growth factor type 1.

Figure 1 illustrates the process of data collection.

### 2.2. Population

The study group comprised 229 children, with 134 boys. The average age at the initiation of therapy was 108 months (9 years). The mean bone age within the study group was 84 months (7 years). At the start of therapy, 186 patients were classified as Tanner stage 1, indicating that puberty had not yet commenced. Additionally, 30 patients were classified as Tanner stage 2, 10 patients as stage 3, and 3 patients as stage 4. The mean birth weight, expressed in SDs, was −3.24, while the mean pretreatment body height, expressed in SDs, was −3.05.

The rhGH dosage is uniform across the country and is within the range of 0.022–0.061 mg/kg/day, preferably 0.036 mg/kg/day.

At the initiation of treatment, the average rhGH dose was 0.031 mg/kg/d. Characteristics of patients at each clinical center enrolled in the study as well as baseline characteristics of the whole study group are presented in Table 1.

### 2.3. Data Collection

Patients qualified for the growth hormone treatment program based on uniform criteria were included in the study. The analysis included (a) birth data of patients defining the diagnosis of SGA (birth weight, birth weight SDs, birth length, birth length SDs); (b) anthropometric data at the start of treatment: age, sex, Ht, SD Ht, growth rate (HV), body weight, insulin-like growth factor 1 (IGF-1) concentration (ng/mL) and rhGH dose (mg/kg/d); (c) anthropometric data at follow-up at 12 and 24 months of treatment (±3 months): Hv, SD Hv, Ht, SD Ht, IGF-1 concentration (ng/mL), and rhGH dose (mg/kg/d).

Anthropometric measurements were obtained during regular follow-up visits at 12 and 24 months (±3 months) following the initiation of treatment. Height was measured three times using a Harpenden stadiometer, which provided accuracy up to ±0.1 cm. The average height was calculated from these measurements. Body weight was measured using certified medical scales with an accuracy of ±100 g.

### 2.4. Data Analyses

The auxological index calculator (developed by U. Smyczynska and P. Smyczynska) was used to convert anthropometric parameters to SDs based on the Polish centile charts of Warsaw children from 2001 [24]. Unsatisfactory response to treatment was determined by the body height difference (∆Ht) between treatment initiation and follow-up at 12 and 24 months, with a threshold of less than 0.3 SDs.

The IGF-1 concentration was measured in laboratories affiliated with the treatment centers. In each patient, irrespective of the treatment center, a fasting blood sample was taken in the morning during the follow-up visit. The IGF-1 determination was performed in the laboratory within 24 h, during which time the material was stored according to laboratory procedures. At each center included in the study, IGF-1 concentration is determined using the immunoassay method.

At the centers in Katowice, Warsaw, and Wroclaw, IGF-1 was measured by random-access immunoassay with enzyme-linked enhanced chemiluminescence (CLIA) using the Siemens IMMULITE 2000 XPi Immunoassay System (Siemens Healthcare Diagnostics Inc. Laboratory Diagnostics 62 Flanders-Bartley Road Flanders, NJ, USA).

In this assay, murine anti-IGF-1 is coated on solid phase beads to act as the capture antibody, while polyclonal rabbit anti-IGF-1 conjugated to alkaline phosphatase serves as the detection antibody. The manufacturer’s instructions include an on-board predilution and acidification step (pHb3.1) to bypass IGFBP interferents, ensuring the separation of IGF-1 and IGFBP-3. Following sample re-neutralization, excess IGF2 is added to block the IGFBP binding sites, preventing re-aggregation of IGF-1 and IGFBP-3 [13,25].

In Bialystok and Szczecin, the patented electrochemiluminescence (ECL) technology method for immunoassays—Elecsys^®^ IGF-1 (COBAS e 411 analyzer, Roche Diagnostics GmbH, Mannheim, Germany)—is used for IGF-1 determination.

According to the manufacturer’s instructions, the methodology is as follows: the complexed antigen in the sample (10 µL) and diluted HCl react to cleave IGF-1 from IGFBP-3 and ALS. During the second incubation, the biotinylated IGF-1 monoclonal antibody and IGF-1 monoclonal antibody (labeled with ruthenium complex) react to form a sandwich-type complex. The streptavidin-coated microparticles facilitate the binding of the complex to the solid phase through the interaction between biotin and streptavidin. Following this, the reaction mixture is transferred to the measuring cell, where the microparticles are magnetically captured on the electrode surface. ProCell/ProCell M is then utilized to remove any unbound substances. By applying a voltage to the electrode, chemiluminescent emission is induced and subsequently measured by a photomultiplier. The results are obtained from a calibration curve specific to the instrument, which is generated through a 2-point calibration and a standard curve provided by the reagent barcode or barcode e [26,27].

There was overlap between reference intervals at the above centers, therefore patients from both centers were analyzed as one group.

In Krakow, IGF-1 levels were determined using a radioimmunoassay for in vitro quantitative measurement of human insulin-like growth factor 1 (IGF-1) in serum (SM-C-RIA-CT from DIAsource ImmunoAssays S.A., Rue du Bosquet, 2 B-1348 Louvain-la-Neuve, Belgium). Activity was measured using a WIZARD 1470 gamma-ray counter from PerkinElmer^®^ (940 Winter Street, Waltham, MA, USA).

To enhance the clinical performance of the assay, DIAsource has incorporated a pretreatment step. In this kit, a specific amount of 125I-labeled IGF-1 is introduced to compete with the IGF-1 present in the sample or calibrator for a fixed number of antibody sites immobilized on the polystyrene tube’s inner wall. Following a 2 h incubation at room temperature with gentle shaking, the competition reaction is completed during the aspiration step. Subsequently, the tubes are washed with 2 mL of washing solution and aspirated. By plotting a calibration curve, the IGF-1 concentrations in the samples can be determined by interpolating the corresponding dose from the calibration curve [28].

The cut-off values for IGF-1 concentrations for both local laboratories and the Italian population were set at 3 percentile and 97 percentile for sex and age. Values < 3rd percentile were considered below normal, while values >97th percentile were considered above normal for age and gender.

In every clinical center enrolled in the study, center-specific laboratory IGF-1 concentration standards were used. Reference intervals used in every GH treatment center are presented in Table 1.

The local standards, to which the results of patients at a particular treatment center were compared, were provided by the laboratory performing the test, based on the standards included in the manufacturer’s specifications for the used test. The result of the IGF-1 determination was compared to the center-specific standard. The norms provided by the manufacturers, and therefore used during the follow-up visits of patients treated with rhGH, are based on healthy subjects. For the purposes of this article, the result of the determination of IGF-1 concentration was compared to the local norm, as well as to the population norm chosen by the authors on the example of the Italian norm (due to the lack of a Polish norm).

To compare reference ranges, the data provided by Bedogni et al. were utilized. In their study, IGF-1 measurements were conducted on 24,403 children (50.6% girls) aged 0 to 18 years using a solid phase, enzyme-labeled chemiluminescent immunometric assay. Age- and sex-specific reference values were generated by employing quantile regression combined with multivariable fractional polynomials [13].

An unsatisfactory response to treatment was characterized by a difference in body height (∆Ht) between treatment initiation and follow-up at 12 and 24 months that was less than 0.3 SDs. Based on their treatment response, patients were categorized into two groups: those with a poor response (∆Ht SDs < 0.3) and those with a good response (∆Ht SDs ≥ 0.3) (Table 2).

### 2.5. Statistical Analyses

Statistical analyses were performed using the R programming and statistical environment (“R environment: a language and environment for statistical computing. R Foundation for Statistical Computing”, Vienna, Austria, version: 4.2.2). Descriptive statistics were performed to describe individual subgroups by providing numbers and percentages of individual subgroups for qualitative data. In order to estimate differences between groups Fisher’s exact test for count data was used. Significance was set at *p* < 0.05.

### 2.6. Ethical and Legal Considerations

This study was carried out in compliance with the Statute of the Department of Pediatrics, Endocrinology, Diabetology, Metabolic Disorders, and Cardiology of Developmental Age (Statute no. WMS-123/01/S/12/2019), which was approved by the Pomeranian Medical University in Szczecin, Poland. It was an observational study conducted in accordance with the guidelines outlined in the Declaration of Helsinki. Prior to the initiation of treatment, the parents of all children receiving rhGH in Poland provided their consent for the treatment.

## 3. Results

### 3.1. The IGF-1 Concentration in Accordance to Local or Population (Italian) Reference Range

At the start of the rhGH treatment program, 185 patients (81%) remained within the normal range for IGF-1 with reference to the local ranges and 215 (94%) with reference to the population (Italian) reference ranges. After 1 year of treatment, with reference to the local norm, 56 patients (24%) presented IGF-1 values > 97th percentile, whereas using the population norm only 8 (3.5%) did so; *p* < 0.001. After 24 months of treatment, the values were: 47 (33%) > 97th percentile by local standard vs. 6 (4.2%) by population standard; *p* < 0.001 (Table 3).

### 3.2. The IGF-1 Concentration in Non-Responding Subgroup (∆Ht SDs < 0.3)

After the first 12 months of treatment 37 patients out of 229 (16%) met the criterion of poor response to treatment defined by ∆Ht SDs < 0.3 and 64 patients out of 144 (44%) after 24 months of treatment. After 12 months of rhGH treatment eight (22%) patients who met the criterion of poor response to treatment presented IGF-1 values were elevated > 97th percentile while assessing with the local reference ranges and three (8.1%) while using the population norms. After 24 months of treatment, the values were: 18 (28%) > 97th percentile by local standard vs. 3 (4.7%) by population standard; *p* < 0.001 (Table 4).

### 3.3. Patients with rhGH Dose Reduction Due to Elevated IGF-1 Concentration Level Based on Local or Italian Reference Range

The rhGH dosing was compared in the same group of patients with reference to the local and population norms. After 12 months, 39 patients (17%) required growth hormone dose reduction, of which, with reference to the local norm, 12 (25%) had elevated IGF-1 levels > 97th percentile and 5 (13%) with reference to the population norm; *p* < 0.05. After 24 months, 35 (25%) patients required dose reduction, of which 23 (66%) had elevated IGF-1 with reference to the local norm and 4 (11%) with reference to the population norm; *p* > 0.05. The group of patients without reduced rhGH dose included those with elevated IGF-1 levels > 97th percentile.

## 4. Discussion

The concentrations of IGF-1 can be influenced by various physiological variables and clinical conditions. Factors such as age, pubertal stage, pregnancy, and extremes of body mass index hold particular significance and should be carefully considered when interpreting results [17]. During childhood and puberty, the IGF-1 concentration undergoes the most substantial changes, while its rate of change slows down with advancing age. To establish normative data, it is crucial to stratify age groups based on well-designed studies and perform statistical analysis on the normative data [18].

The clinician interpreting the IGF-1 results should be aware that chronic diseases such as cirrhosis, liver failure, and renal failure or taking estrogen medication affect the outcome [29].

IGF-1 value is also affected by the technique of collection of blood samples, storage of the material, and the time from blood sampling to the assay. It is postulated that blood samples should be processed within 2 h to avoid an artifactual increase in results [17]. Special attention should therefore be paid to possible prelaboratory error due to prolonged processing of the material. Therefore, it seems impossible to organize one central laboratory to carry out IGF-1 assays for all patients treated with growth hormone in the case of a country such as Poland. In blood, IGF-1 is present in a complex with insulin-like growth factor binding protein type 3 (IGFBP-3) and acid labile subunit (ALS). In order to obtain the most reliable measurements, this complex should be broken down before the assay is performed. In each of the methods used in treatment centers enrolled in this study, IGF-1 is released from binding proteins [25,26,28].

As part of the treatment of short stature in SGA children, pharmacological doses of rhGH are used, higher than in the treatment of somatotropin hypopituitarism. One reason for this is that SGA patients are a very heterogeneous group and the cause of short stature and lack of catch-up growth is attributed to, among other things, already reduced IGF-1 concentrations from the fetal period or partial resistance to GH, rather than a deficiency of endogenous growth hormone per se [30]. The use of pharmacological doses of hormone poses the risk of developing more side effects. At the same time, it is known that an increase in IGF-1 levels is observed in good response to GH treatment. When analyzing groups of patients treated for short stature of various etiologies, it can be observed that many of them do not respond as expected to treatment. Reasons for this may include a reduction in the dose of growth hormone, secondary to elevated IGF-1 concentrations above the reference value. This makes it all the more relevant that, depending on the accepted IGF-1 standard, a different proportion of patients require dose reduction (Table 5).

The authors of a previous article covering the same population noted that during follow-up most patients maintained IGF-1 levels within the normal range [12]. This observation did not coincide with clinical experience. The researchers’ attention was drawn to the fact that there was a higher proportion of patients who required rhGH dose modification due to elevated IGF-1 levels in daily clinical practice than some published results indicated. This difference is attributable to the fact that, in the absence of standardized pediatric IGF-1 concentration norms, the population norms described by Bedogni et al. in the Italian population were used for the previous analysis in order to standardize the analyzed data. After reviewing the IGF-1 values in relation to the local norms used in each center, presented by the laboratory performing the IGF-1 assay, it became clear that there were statistically significant differences between the standards.

The authors of this article concluded that it is difficult to standardize the management of GH therapy in a country as large as Poland. One of the reasons might be that right now, due to the open market and the fact that laboratories supporting GH treatment centers independently choose the IGF-1 measurement method, it is not possible to designate only one method for the entire country. The suggestion to choose one manufacturer for the whole country is worth emphasizing in recommendations.

The IGF-1 levels at the baseline for age and sex with reference to the local norm were observed in 81% of patients, while when using the Italian population norm, 94% of patients were defined as having normal IGF-1 levels. In general, while using the local reference ranges, in 24% of patients after 12 months and in 33% of patients after 24 months of rhGH treatment elevation of IGF-1 above the 97th percentile was observed. The values obtained using local standards are higher than those presented by Cabrol et al. [31]. On the other hand, with usage of the Italian norms the percentages were: 3.5% after 12 months and 4.2% after 24 months.

Bearing in mind that the assessment of IGF-1 levels determines the dosage and possible exclusion of the patient from the therapeutic program, it seems extremely important to analyze these norms.

Other researchers have attempted to create IGF-1 reference values for pediatric populations [18,32]. It seems justified to attempt to create analogous values dedicated to both the general pediatric population in Poland and to children treated with rhGH, including those born SGA. It has been repeatedly demonstrated that rhGH dosing based on IGF-1 concentrations compared to dosing based on the patient’s body weight results in a better response to treatment and, consequently, achievement of the expected final height [9,10] Although some of the cited studies included a group of children with idiopathic short stature (ISS), it can be assumed that SGA children were also included. It has been postulated that this method of rhGH dosing and therapy management allows individualization of treatment, especially in the case of possible partial resistance to rhGH. In their study, Park et al. showed that guiding therapy and maintaining elevated IGF-1 concentrations (even up to +3 SDs for sex and age) results in a good response to treatment while also demonstrating the safety of therapy. After dose reduction/termination of treatment, IGF-1 concentrations returned to the expected range. Higher IGF-1 concentrations were associated with higher dose of rhGH and therefore better growth [33].

In our study group, 39 children (17%) required dose reduction after 12 months of treatment and 35 children (25%) after 24 months. These values are similar to the data presented by Cabrol et al. [31]. Depending on the reference value adopted, a statistically significantly different proportion of patients had IGF-1 levels > 97th percentile. The reference ranges based on Bedgoni et al.’s work are more tolerable and may be more favorable for the patient, assuming that GH dosing is based only on IGF-1 concentrations. At the same time, it should be noted that the group of patients without dose reduction also included patients with elevated IGF-1. This indicates that the IGF-1 value is not the only criterion on which rhGH dosing depends.

In the course of collecting data for this publication and from the authors’ experience, an increased number of side effects if elevated IGF-1 levels persisted was not observed. The reduction of the growth hormone dose was mostly due to the terms of the nationwide rhGH treatment program. It would be optimal to create IGF-1 reference ranges based on the Polish population and preferably dedicated to specific subgroups treated with rhGH. Another parameter assessed at the follow-up visit and which determines the continuation or modification of treatment is body height and, consequently, the assessment of growth. One of the criteria used is the difference in body height expressed in SDs. A ∆Ht SD < 0.3 is considered a criterion for a poor response [34]. In our study group, considering only patients with ∆Ht SDs < 0.3 after 12 months of rhGH treatment, eight (22%) patients presented IGF-1 values that were elevated > 97th percentile while assessing with the local reference ranges and three (8.1%) while using the population norms. After 24 months of treatment, the values were: 18 (28%) > 97th percentile by local standard vs. 3 (4.7%) by population standard. An increase in IGF-1 is a prognostic indicator of response to treatment. It may be observed that children have IGF-1 concentrations in the normal range during treatment and respond poorly. It should be noted that there are many factors influencing treatment, such as the advancement of bone age, the presence of dysmorphic features which may be indicative of a genetically determined disorder (short statue syndrome), etc. We have discussed this topic in our previous paper [12].

Recombinant human growth hormone has been extensively utilized for the treatment of short stature in children since 1985. Indications for treatment include somatotropic hypopituitarism, Turner syndrome, SGA, and others. Due to its biological activity, consisting of anti-apoptotic and mitogenic effects, the involvement of IGF-1 in the potential development of malignancy has been extensively studied. It has been demonstrated in mouse models and in vitro human tumor tissues that elevated IGF-1 slightly increases the risk of malignancy, but this was not supported in human models. On the basis of numerous meta-analyses and systemic reviews, it is postulated that elevated IGF-1 due to the biological effects of rhGH does not increase the risk of malignancy during and after growth hormone treatment. Treatment with rhGH is considered safe [35,36]

In addition, metabolic complications in the form of lipid or carbohydrate disturbances are mentioned as side effects of excessive IGF-1 concentrations [37].

Xu et al., in their meta-analysis, demonstrated that abnormalities in glucose metabolism and insulin sensitivity begin during preadolescence in children with SGA. Furthermore, the study revealed that these metabolic abnormalities may progressively worsen over time. There is a potential association between SGA and the onset of diabetes at an early age [38].

In light of these data, the safety of GH treatment, whose spectrum of action includes anabolic and hyperglycemic effects, has been debated. However, it has been shown that HOMA-IR increases during the first year of therapy but stabilizes throughout therapy and normalizes after the end of therapy [39].

At the same time, the relationship between rhGH dose and response to treatment is very well documented—the higher the initial dose, the better the therapeutic effect [40]. Therefore, with the patient’s best interest in mind, it would be advisable to try to avoid rhGH dose reduction. In our analysis, it is evident that, depending on the adopted standard, a statistically significantly different proportion of patients was found above the 97 percentile for sex and age in the context of IGF-1 levels.

Another factor influencing the response to treatment is the age at the start of therapy. The mean age of treatment initiation for our study group was 108 months (9 years). As we showed in the previous article, this is later than the recommended 4 years for the Polish population, but comparable to the age reached in other European centers [12,41,42]. The identification of short stature patients born SGA should be pursued as early as possible as one of the factors that can significantly affect therapeutic outcomes. It has been proven that only the free fraction of IGF-1 shows bioactivity. This makes the triple complex, by reducing the availability of IGF-1 to target cells, the main reservoir of IGF-1 in the circulation, as well as the main regulator of its bioavailability and bioactivity [43]. In view of this, the question arises as to whether IGF-1 concentration is a good marker of response to growth hormone treatment, or whether an assessment of bioactive IGF-1 would be better. In their study, Wegmann et al. undertook an analysis of bioactive IGF-1 concentration compared to total IGF-1 concentration in SGA children treated with rhGH. Despite elevated total IGF-1 concentrations, the majority of children exhibited bioactive IGF concentrations within the normal range, as demonstrated by their study.

This may be an argument for the safety of the therapy and condoning the maintenance of a higher dose of rhGH despite elevated IGF-1 [44].

Given all of the above, it appears that the temporary maintenance of elevated IGF-1 levels above reference ranges is safe and beneficial for the patients.

In view of the growing group of children being treated for short stature, it would be advisable to review the methods of IGF-1 measurement and to consider standardizing them across the country, which would enable therapy to be administered fairly. It also seems reasonable to try to create IGF-1 reference ranges dedicated to specific subgroups of children treated with rhGH. This requires further research.

### Limitations of Work

One study limitation is the size of the group—although, as the reviewer of our previous paper pointed out, there are still not many reports covering rhGH therapy in SGA children.

Another study limitation is the potential heterogeneity of the SGA group, which may affect IGF-1 concentrations and consequently the response to treatment. However, low baseline IGF-1 concentrations, secondary to, e.g., a low body weight, were not reported.

A limitation of the study is the fact that IGF-1 determination was performed in different laboratories with equipment from different companies and with different testing methods. However, data were compared to center-specific standards.

The work was also limited by its observational nature.

## 5. Conclusions

The different methods used to determine IGF-1 concentration and the different IGF-1 reference ranges result in a significant proportion of rhGH-treated children with elevated IGF-1 concentration and experiencing dose reductions, which may negatively affect growth rate.

It would be optimal to determine IGF-1 levels in all children using the same method and in the same laboratory, but this is difficult to achieve in a country with a population similar to the Polish one.

In view of the increasing number of short stature patients being treated with growth hormone, it would appear reasonable to create IGF-1 standards dedicated to each of the subgroups provided with rhGH treatment.

## Figures and Tables

**Table 1:** Selected background characteristics of the study group.

Characteristic	All Mean (SD)	Szczecin + Bialystok Mean (SD)	Katowice Mean (SD)	Warsaw Mean (SD)	Wroclaw Mean (SD)	Krakow Mean (SD)
*n*	229	64	55	41	32	37
Gender						
F	95	27	24	15	10	19
M	134	37	31	26	22	18
Age at onset of treatment [months]	108 (35)	116 (39)	106 (35)	107 (30)	104 (37)	105 (34)
Tanner stage at onset of treatment						
1	186	52	46	32	27	29
2	30	8	6	7	3	6
3	10	2	3	2	2	1
4	3	2	0	0	0	1
Birth weight, SDs	−3.24 (1.17)	−3.27 (1.16)	−3.32 (1.28)	−2.77 (0.65)	−3.54 (1.20)	−3.34 (1.33)
Birth length, SDs	−1.00 (1.54)	−0.75 (1.60)	−1.17 (1.39)	−0.63 (1.21)	−1.24 (1.60)	−1.36 (1.83)
Ht0, SDs	−3.05 (0.79)	−2.92 (0.59)	−3.25 (0.97)	−2.47 (0.41)	−3.30 (0.72)	−3.39 (0.83)
BMI0	14.73 (2.05)	14.42 (2.07)	15.15 (2.39)	15.18 (1.63)	14.84 (1.88)	14.11 (1.97)
BA [months]	84 (39)	94 (41)	78 (37)	86 (36)	76 (40)	79 (36)
IGF-1-0 [ng/mL]	142 (84)	126 (71)	135 (72)	154 (84)	125 (94)	185 (98)
IGF-1-12 [ng/mL]	299 (164)	317 (169)	248 (126)	243 (107)	273 (150)	431 (193)
IGF-1-24 [ng/mL]	342 (184)	378 (223)	264 (135)	322 (139)	300 (135)	511 (188)
GH0 dose [mg/kg/d]	0.031 (0.005)	0.034 (0.004)	0.025 (0.003)	0.028 (0.002)	0.035 (0.000)	0.033 (0.005)
GH12 dose [mg/kg/d]	0.032 (0.005)	0.035 (0.003)	0.029 (0.003)	0.029 (0.004)	0.035 (0.000)	0.035 (0.004)
GH24 dose [mg/kg/d]	0.033 (0.004)	0.033 (0.006)	0.032 (0.004)	0.031 (0.004)	0.035 (0.000)	0.034 (0.004)
∆Ht SDs 12-0	0.57 (0.33)	0.57 (0.31)	0.46 (0.31)	0.54 (0.29)	0.73 (0.32)	0.63 (0.42)
∆Ht SDs 24-12	0.38 (0.33)	0.40 (0.41)	0.43 (0.30)	0.18 (0.16)	0.39 (0.28)	0.50 (0.38)
∆Ht SDs 24-0	0.97 (0.50)	0.94 (0.49)	0.90 (0.46)	0.78 (0.28)	1.11 (0.51)	1.25 (0.62)

Ht—height; BA—bone age; IGF-1—insulin-like growth factor 1; GH—growth hormone; GH0—growth hormone dose at baseline; GH12—growth hormone dose after 12 months; GH24—growth hormone dose after 24 months.

**Table 2:** Local reference ranges of clinical centers enrolled in the study.

IGF-1 [ng/dL]										
Center	Bialystok + Szczecin		Katowice		Warsaw		Wroclaw		Krakow	
Sex	Male	Female	Male	Female	Male	Female	Male	Female	Male	Female
Age [years]										
1	11.8–96.4	18.7–104	15–189	15–272	15–129	18.2–172	55–327	55–327	32–339	45–361
2	13.9–104	26.1–128	15–189	15–272	15–129	18.2–172	51–303	51–303	32–339	45–361
3	18.9–116	34.2–155	15–189	15–272	15–129.1	35.4–232	49–289	49–289	47–287	42–276
4	26.8–134	43.2–185	47–231	55–248	22–208	35.4–232	49–283	49–283	47–287	42–276
5	36.6–156	53.0–216	47–231	55–248	22–208	35.4–232	50–286	50–286	47–287	42–276
6	47.1–184	63.6–250	47–231	55–248	40.1–255	56.9–277	52–297	52–297	75–311	59–297
7	57.5–216	75.0–286	55–222	80–233	40.1–255	56.9–277	57–316	57–316	75–311	59–297
8	67.5–254	87.3–324	55–222	80–233	40.1–255	56.9–277	64–345	64–345	75–311	59–297
9	76.9–296	99.9–363	55–222	80–233	68.7–316	118–448	74–388	74–388	85–553	188–515
10	85.7–343	112–398	95–315	96–545	68.7–316	118–448	88–452	88–452	85–553	188–515
11	93.9–392	123–427	95–315	96–545	143–506	170–527	111–551	111–551	85–553	188–515
12	101–434	132–451	95–460	147–549	143–506	170–527	143–693	143–693	139–727	214–753
13	108–467	140–468	95–460	147–549	177–507	191–496	183–850	183–850	139–727	214–753
14	115–489	146–480	211–512	208–444	177–507	191–496	220–972	220–972	139–727	214–753
15	120–501	151–485	211–512	208–444	173–414	190–429	237–996	237–996	123–1016	210–1064
16	125–503	154–485	57–426	176–429	173–414	190–429	226–903	226–903	123–1016	210–1064
17	129–495	156–479	57–426	176–429	173–414	190–429	193–731	193–731	123–1016	210–1064
18	132–476	156–466	57–426	176–429	173–414	190–429	163–584	163–584	135–1276	70–758
Method:	ECL Elecsys^®^ IGF-1 (COBAS e 411 analyzer, Roche)		CLIA (Siemens IMMULITE 2000 XPi Immunoassay System)		CLIA (Siemens IMMULITE 2000 XPi Immunoassay System)		CLIA (Siemens IMMULITE 2000 XPi Immunoassay System)		SM-C-RIA-CT (DIAsource ImmunoAssays S.A.)	

**Table 3:** IGF-1 concentration in accordance with local or Italian reference range.

IGF-1 at Baseline	Local Reference Ranges, *n* = 229 ^1^	Population Reference Ranges, *n* = 229 ^1^	*p*-Value ^2^
Below norm	42 (18%)	14 (6.1%)	<0.001
In norm	185 (81%)	215 (94%)	
Above norm	2 (0.9%)	0 (0%)	
IGF-1 after 12 months	Local reference ranges, *n* = 229 ^1^	Population reference ranges, *n* = 229 ^1^	<0.001
Below norm	1 (0.4%)	1 (0.4%)	
In norm	172 (75%)	220 (96%)	
Above norm	56 (24%)	8 (3.5%)	
IGF-1 after 24 months	Local reference ranges, *n* = 144 ^1^	Population reference ranges, *n* = 144 ^1^	<0.001
Below norm	1 (0.7%)	1 (0.7%)	
In norm	96 (67%)	137 (95%)	
Above norm	47 (33%)	6 (4.2%)	

^1^ *n* (%); ^2^ Fisher’s exact test.

**Table 4:** IGF-1 concentration among patients who did not respond to treatment (∆Ht SDs < 0.3).

IGF-1 after 12 Months	Local Reference Ranges, *n* = 37 ^1^	Population Reference Ranges, *n* = 37 ^1^	*p*-Value ^2^ 0.2
Below norm	0 (0%)	0 (0%)	
In norm	29 (78%)	34 (92%)	
Above norm	8 (22%)	3 (8.1%)	
IGF-1 after 24 months	Local reference ranges, *n* = 64 ^1^	Population reference ranges, *n* = 64 ^1^	<0.001
Below norm	1 (1.6%)	1 (1.6%)	
In norm	45 (70%)	60 (94%)	
Above norm	18 (28%)	3 (4.7%)	

^1^ *n* (%); ^2^ Fisher’s exact test.

**Table 5:** Patients with rhGH dose reduction due to elevated IGF-1 concentration level based on local or population reference range.

	Local Reference Ranges		Population Reference Ranges		*p*-Value ^1^
After 12 months *n* = 227	With rhGH dose reduction *n* = 39	Without rhGH dose reduction *n* = 188	With rhGH dose reduction *n* = 39	Without rhGH dose reduction *n* =188	<0.05
Below norm	0	0	0	0	
In norm	27 (75%)	144 (77%)	34 (87%)	185 (98%)	
Above norm	12 (25%)	44 (23%)	5 (13%)	3 (2%)	
After 24 months *n* = 140	With rhGH dose reduction *n* = 35	Without rhGH dose reduction *n* = 105	With rhGH dose reduction *n* = 35	Without rhGH dose reduction *n* = 105	0.67
Below norm	0	0	0	0	
In norm	12 (34%)	81 (77%)	31 (89%)	103 (98%)	
Above norm	23 (66%)	24 (23%)	4 (11%)	2 (2%)	

^1^ Fisher’s exact test.

## Data Availability

The data presented in this study are available on request from the corresponding author. The data are not publicly available due to ethical restrictions.
